# Modeling of the Influence of Input AM Parameters on Dimensional Error and Form Errors in PLA Parts Printed with FFF Technology

**DOI:** 10.3390/polym13234152

**Published:** 2021-11-27

**Authors:** Carmelo J. Luis-Pérez, Irene Buj-Corral, Xavier Sánchez-Casas

**Affiliations:** 1 Engineering Department, Arrosadia Campus, Public University of Navarre (UPNA), 31006 Pamplona, Spain; cluis.perez@unavarra.es; 2 Department of Mechanical Engineering, Barcelona School of Engineering (ETSEIB), Universitat Politècnica de Catalunya-Barcelona Tech (UPC), 08028 Barcelona, Spain; xasc95@gmail.com

**Keywords:** additive manufacturing, surface roughness, FFF, ANFIS, modeling, desirability

## Abstract

As is widely known, additive manufacturing (AM) allows very complex parts to be manufactured with porous structures at a relatively low cost and in relatively low manufacturing times. However, it is necessary to determine in a precise way the input values that allow better results to be obtained in terms of microgeometry, form errors, and dimensional error. In an earlier work, the influence of the process parameters on surface roughness obtained in fused filament fabrication (FFF) processes was analyzed. This present study focuses on form errors as well as on dimensional error of hemispherical cups, with a similar shape to that of the acetabular cup of hip prostheses. The specimens were 3D printed in polylactic acid (PLA). Process variables are nozzle diameter, temperature, layer height, print speed, and extrusion multiplier. Their influence on roundness, concentricity, and dimensional error is considered. To do this, adaptive neuro-fuzzy inference systems (ANFIS) models were used. It was observed that dimensional error, roundness, and concentricity depend mainly on the nozzle diameter and on layer height. Moreover, high nozzle diameter of 0.6 mm and high layer height of 0.3 mm are not recommended. A desirability function was employed along with the ANFIS models in order to determine the optimal manufacturing conditions. The main aim of the multi-objective optimization study was to minimize average surface roughness (Ra) and roundness, while dimensional error was kept within the interval $\left|Dimensional\;Error\right|\leq 0.01$. When the simultaneous optimization of both the internal and the external surface of the parts is performed, it is recommended that a nozzle diameter of 0.4 mm be used, to have a temperature of 197 °C, a layer height of 0.1 mm, a print speed of 42 mm/s, and extrusion multiplier of 94.8%. This study will help to determine the influence of the process parameters on the quality of the manufactured parts.

## 1. Introduction

Extrusion printing processes such as fused filament fabrication (FFF) allow printing parts with complex shapes without high costs if low-cost machines are employed. However, surface finish, form error, and dimensional quality of the printed parts are not excellent [1,2]. Additive manufacturing (AM) processes have been widely used in different sectors such as the automotive, electronics, and medical sectors. In the latter case, AM has been used to manufacture, among others, surgical guides, phantoms, implants, etc. [3]. Specifically, prostheses require a smooth surface in those areas which will be in contact with sliding elements. They also require low form errors, so that they are as similar as possible to the original bone. For example, it is important to have a low concentricity error in order to ensure a good fit between the different elements and good operation of the prosthesis. As for dimensional error, in the specific case of hip arthroplasty, in order to reduce costs, standardized prostheses of different sizes are currently used that suppliers offer on the basis of anthropomorphic data and market needs. However, in those patients who exceed the standard ranges, between two sizes or with special requirements due to diseases or genetic problems, the surgical process becomes much more complex and expensive [4].

The effect of FFF printing parameters on dimensional error and on form errors of printed parts has been previously studied in the literature. For example, Rahman et al. [5] studied the dimensional deviations in X, Y, and Z of acrylonitrile–butadiene–styrene (ABS) printed parts. They recommend low bed temperature, low nozzle temperature, high print speed, medium infill, low layer thickness, and a low number of shells in order to reduce dimensional error. Ceretti et al. [6] 3D printed polycaprolactone (PCL) parts with rectilinear grid structure. They observed that the nominal size of pores was the most influential parameter on the extruded diameter of the filament, while the head type greatly influenced the resulting height of the pores. Nancharaiah et al. [7] found that small thickness layer and large bead width improve part accuracy. Raster angle showed a slight effect on part accuracy. In ABS parts, Pennington et al. [8] found that the most influential factors on dimensional accuracy were part size, the location of the part in the work envelope, and the envelope temperature. Garg et al. [1] showed that the dimensional accuracy of ABS parts did not vary significantly when they were submitted to cold vapor treatment. Akbaş et al. [9] observed higher dimensional accuracy in polylactic acid (PLA) than in acrylonitrile butadiene styrene (ABS) printed parts. As a general trend, for PLA, the strip width increased with increasing temperature and decreased with increased feed rate.

Regarding form errors of 3D printed parts, Maurya et al. [10] studied the effect of infill pattern, layer thickness, build orientation, and infill density on flatness and cylindricity of 3D printed components. The most appropriate printing conditions were found to be layer thickness 100 μm, linear infill pattern, 45° orientation, and 20% infill density. Knoop et al. [11] investigated the cylindricity of inner and outer cylinders, with different diameters between 3 and 80 mm. They observed better roundness in the XY-plane than in the Z direction. Highest mean roundness values up to 0.27 mm were reported for holes and up to 0.32 mm for cylinders, corresponding to highest diameter of 80 mm. Saqib and Urbanic [12] evaluated flatness, perpendicularity, and cylindricity of cope and drag casting elements. They observed that the shape of the parts had a greater influence on their form errors than the printing parameters. Ollison and Beriso [13] studied the effect of build orientation, diameter, and printhead life on cylindricity of 3D printed parts. Build orientation was the most influential parameter, a 90° angle having the highest cylindricity error (part rotated 90° about the X axis). Paul and Anand [14] proposed a voxel-based approach to optimize the flatness and cylindricity of parts, while reducing the utilization of support materials. Spindola Filho et al. [15] found cylindricity values up to 0.140 mm being higher on the external surface than on the internal surface of cylindrical shapes.

As for concentricity, Boejang et al. reported different values in FDM processes, depending on the machine employed, ranging between 0.1622 and 0.5784 mm [16]. Concentricity values up to 0.2229 mm were obtained by Abdelrhman [17]. Spindola Filho et al. [15] found concentricity values up to 0.090 mm, being print speed the most influential factor.

In addition, dealing with the application of artificial neural networks (ANN) and adaptive neuro-fuzzy inference systems (ANFIS) for modelling manufacturing processes, several studies can be found in the literature. However, the application of these soft computing techniques for modelling input parameters in additive manufacturing process has been employed to a lesser extent. Among the recent published studies, it is worth mentioning that of Deswal et al. [18] where response surface methods and ANNs are employed for optimization of input process parameter in order to improve the precision of FFF 3D printed parts. Likewise, Noriega et al. [19] employed an ANN for improving the accuracy of FDM parts. The ANN was used to determine the optimal dimensional values for the CAD model. In another study Padhi et al. [20] employed a Mamdani Fuzzy Inference Systems (FIS) along with the Taguchi method to optimize the extrusion process for the manufacturing of acrylonitrile-butadiene-styrene (ABSP 400) parts. In the study from Peng et al. [21] the authors employed response surface method combined with FIS and genetic algorithm (GA) in order to predict dimensional errors, warp deformation, and build time in FFF.

Some other properties dealing with FFF additive manufacturing technique, have also been studied in recent years. For example, it is worth mentioning the study of Nasiri et al. [22] where a review of applications of machine learning to predict mechanical behavior of 3D printed parts is presented or that of Trivedi et al. [23], where tensile strength is predicted by using Taguchi design of experiments and fuzzy logic. On the other hand, in the study of Rajpurohit et al. [24], an ANFIS is used to predict the tensile strength of FFF printed parts. An ANFIS model is also employed to predict surface roughness, build time, and compressive strength with respect to changes in FFF in Sai et al. [25].

Further examples of the application of fuzzy logic can be found in the study from Xia et al. [26] where surface roughness obtained in wire arc additive manufacturing is predicted by using an ANFIS, that of Sahu et al. [27] where a Mamdani FIS combined with Taguchi method is employed to improve the dimensional accuracy of FFF ABSP 400 parts, and that of Saleh et al. [28] where building orientation, layer thickness, and slurry impact angle are used as input variables in an ANFIS for modelling the effect of slurry impacts on polylactic acid processed by fused deposition modeling or that of or that of Mensah et al. [29] where an ANFIS in employed for flammability parameter modeling among many others.

In a previous work, hemispherical cups were 3D printed in polylactic acid (PLA), with different printing conditions, according to a fractional factorial design. ANFIS models were obtained for different surface roughness parameters measured on hemispherical surfaces that are similar to those of the acetabular part of hip prostheses [30]. In this present work, dimensional error and form errors of FFF 3D printed spherical parts are addressed. Unlike flat surfaces, 3D printed curved surfaces have hardly been studied in the literature regarding dimensional and form errors. When manufacturing a customized prosthesis (for example, to replace the acetabulum), it is important to ensure dimensional accuracy. In addition, form errors such as roundness or concentricity should be reduced in order to ensure the correct performance of the hip joint. ANFIS models are applied to dimensional error and roundness error (measured both on the internal and on the external surface of the parts), as well as to concentricity between both spherical surfaces. Moreover, the methodology and the desirability function proposed by Luis-Pérez [31] is used in this present study to obtain the optimal values of the manufacturing conditions in order to simultaneously minimize the roundness and the average surface roughness and at the same time keeping the dimensional error within a specified tolerance. The results of this work will help to select appropriate 3D printing conditions when obtaining curved surfaces.

## 2. Materials and Methods

In this section, the process that was used to manufacture the parts is explained, as well as the measurement and analysis methodology that were employed.

### 2.1. Printing Process

**Figure 1:**
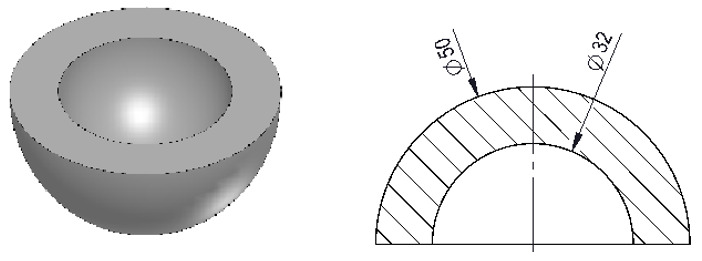
Shape and dimensions of the specimens.

The samples were printed with a Sigma R19 printer from BCN3D Technologies (Gavà, Spain), with white polylactic acid (PLA) filament of 2.85 mm diameter, from BCN3D Technologies. The samples have the shape of hemispherical cups, which are similar to the prostheses that are used to replace the acetabula in hip prostheses. Internal dimeter of 32 mm and external diameter of 50 mm were selected, which are common dimensions of prostheses [32] (Figure 1 shows).

**Figure 2:**
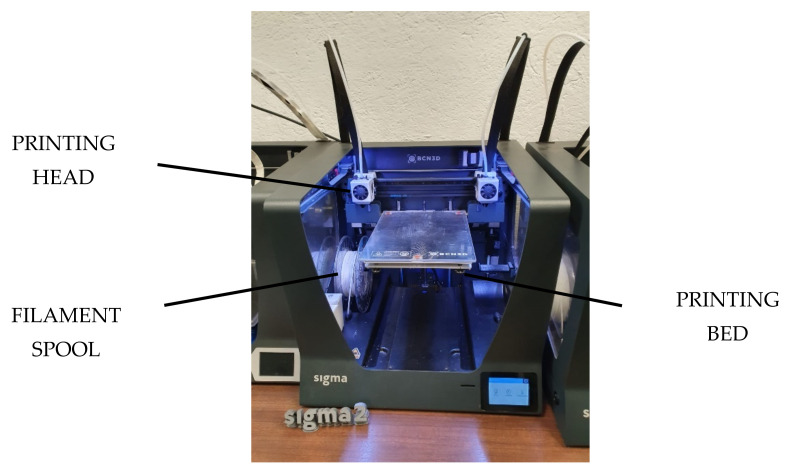
Sigma R19 from BCN3D.

The hemispherical cups were printed in a Sigma R19 machine from BCN3D as shown in Figure 2, with infill value of 20% and shell thickness of 1.2 mm. Printing bed temperature was 65 °C. Printing supports were required, in PLA material. Further details of the printing process are provided in a previous work [30].

The experiments were defined according to a fractional factorial design 2^5-1^, with 5 variables and 2 levels. Selected variables are as follows [30]: Nozzle diameter (ND) between 0.4 and 0.6 mm, temperature (T) between 195 and 205 °C, layer height (LH) between 0.1 and 0.3 mm, print speed (PS) between 30 and 50 mm/s, and extrusion multiplier between 93 and 97%.

The selected responses are shown in Table 1. Both roundness and concentricity are defined in the UNE-EN-ISO 1101:2017 standard [33].

### 2.2. Determination of the Dimensional Error

**Figure 3:**
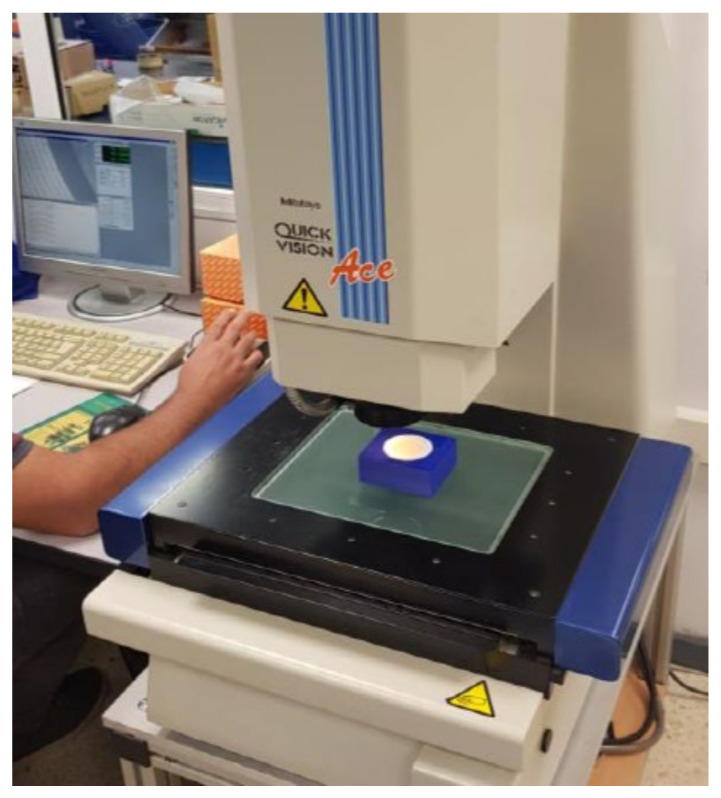
Measurement process of dimensional error.

Dimensional error was measured with a Mitutoyo Quick Vision Ace Equipment. Both the external and the internal diameter of the hemispherical cups were measured, on the base of the hemispherical cups as shown in Figure 3. The specimens were placed on a 3D printed support.

The equipment shown in Figure 3 has a platform on which the support and the part are placed, as well as a head with a lens with 2X magnification. The software allows a digitally magnified and focused image of the part’s surface to be obtained. The platform moves along the XY plane, and its displacement is measured. In order to determine the dimensional error, first the platform is moved so that by clicking on the image six points are obtained along each circumference (external or internal). Then, the software uses these six points to calculate the diameter and the location of the center point of the measured circumference.

Dimensional error is then calculated as the difference between the measured value and the theoretical nominal value of a certain dimension. For this reason, in this case dimensional error takes positive values when the measured diameter is higher than the nominal diameter (and it takes negative values when the measured diameter is lower than the nominal diameter).

### 2.3. Determinación of Roundness

**Figure 4:**
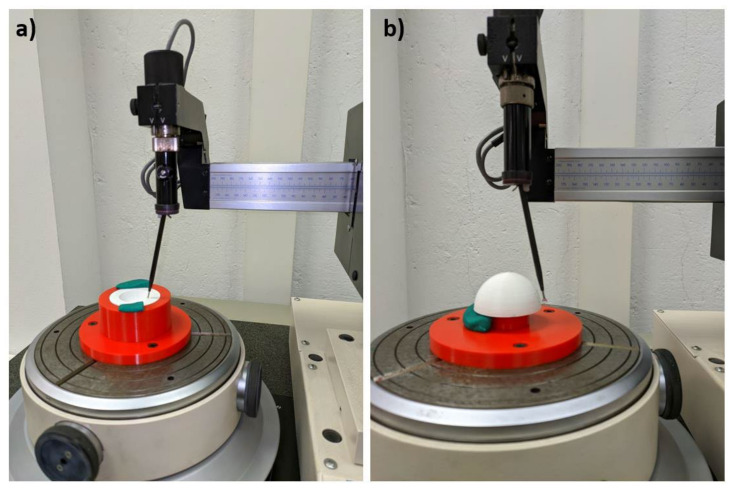
Measurement process of roundness on: (**a**) the internal surface, (**b**) the external surface.

Roundness tolerance measures how far a measured circumference is from an ideal circumference and, according to ISO 1101 standard [33], it is defined as the distance between two ideal circumferences (inscribed and circumscribed of the measured circumference) which are defined on a perpendicular plane to the measured circumference. Roundness was determined with a Taylor Hobson Talyrond 252 measuring machine (as shown in Figure 4). This machine has a spherical point with 16 nm resolution, which can measure roundness errors below 1 µm. It only moves along the Z axis, with high precision, while the part, which is located on a circular platform, rotates. It was necessary to design two different 3D print supports, to fix the parts to the platform of the machine, in order to measure roundness of the internal and of the external surface of the parts, respectively.

### 2.4. Determination of the Concentricity

According to ISO 1101 standard [33], concentricity is defined as the difference between the measured center of a certain measured circumference and a theoretical reference center. In this case, the center of a circumference measured on the internal surface of the hemispherical cups is taken as the reference center, from which concentricity is defined. Concentricity was determined from the data of the position of the circumferences’ centers that were measured with the Mitutoyo Quick Vision Equipment in order to obtain the dimensional error (see Section 2.2).

### 2.5. ANFIS Modellin

A zero-order Sugeno FIS is employed by using the Fuzzy Logic Toolbox™ of Matlab^TM^2020a (Natick, MA, USA) [34] where Gaussian membership functions were employed for fuzzification of the independent variables. Likewise, Equation (1) shows the product implication method and Equation (2) shows the output of the Sugeno system [34,35,36].

(1) $$\lambda_{j}\left(x\right)=AndMethod\left\{\mu_{1}\left(x_{1}\right),\ldots ,\mu_{n}\left(x_{n}\right)\right\}$$

(2) $$\left\{Output\right\}=\frac{\sum_{j=1}^{Number\;of\;rules}\lambda_{j}\times z_{j}}{\sum_{j=1}^{Number\;of\;rules}\lambda_{j}}$$

where the parameters analyzed in this present study are the dimensional error, the roundness error, and the concentricity, which have been defined in the previous section.

## 3. Results and Discussion

This section shows the results obtained when applying the ANFIS for modelling the above-mentioned dimensional parameters. As previously mentioned, the form errors of the manufactured parts were measured inside and outside of the hemispherical cups made of PLA material that were printed using FFF technology. Table 2 presents the form error results for both the internal and the external surfaces of the specimens.

Most experiments show positive dimensional error on the external wall of the parts, showing that the diameter of the printed parts is higher than the theoretical one. Conversely, dimensional error is mainly negative in the internal wall of the parts, indicating that diameters are smaller than expected. This suggests an excess of material in the printed parts with respect to the original geometry of the hemispherical cups. The highest dimensional error value of 0.314 mm was obtained on the external wall of experiment 15, corresponding to low nozzle diameter, high temperature, high layer height, high speed and low extrusion multiplier. Lowest dimensional error was found in experiment three, obtained with low nozzle diameter, high temperature, low layer height, low print speed and low extrusion multiplier. In an earlier paper, higher relative dimensional errors between 0.82% and 2.60% were reported in prismatic porous samples [37]. In this present work, lower relative dimensional errors were obtained, ranging between 0.036% and 0.625%. This can be attributed to the fact that, in this work, dimensional errors have been measured at the base of the printed samples, corresponding to the first layer of the parts, which is directly deposited on the printing bed. Thus, it is less likely to suffer deformation than other layers. Equbal et al. [38] obtained relative error values of up to 0.300% in length, 0.628% in width, and 3.143% in thickness in the ABS material. Spindola Filho et al. [15] found cylindricity values up to 0.140 mm in ABS parts.

Roundness values range between 0.089 mm for experiment 3 and 0.297 mm for experiment 6. Similar values of 0.292 mm were reported by Maurya et al. in PLA parts [39]. Sajan et al. [40] studied circularity of parts that were printed on different planes. For the plane XY, on which the parts of the present study were printed, a maximum circularity value of 0.401 mm is reported.

Concentricity ranges between 0.048 mm (average value for experiment 17, central point) and 0.272 mm for experiment 6. Rupal et al. [41] obtained similar average concentricity values of 0.300 mm in ABS, PLA, and magnetic-PLA parts.

### 3.1. Models for Dimensional Error

**Figure 5:**
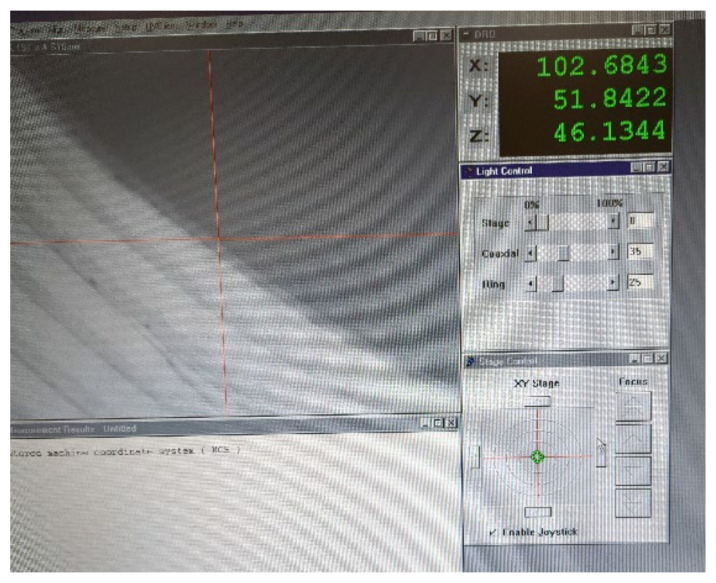
Example of an image obtained with the vision machine.

Figure 5 corresponds to an image of the vision machine showing the base (flat surface) of a hemispherical sample, where both the diameter of the external and of the internal circumference are measured, as well as the location of their center points. From the position of the center points the concentricity error was measured.

**Figure 6:**
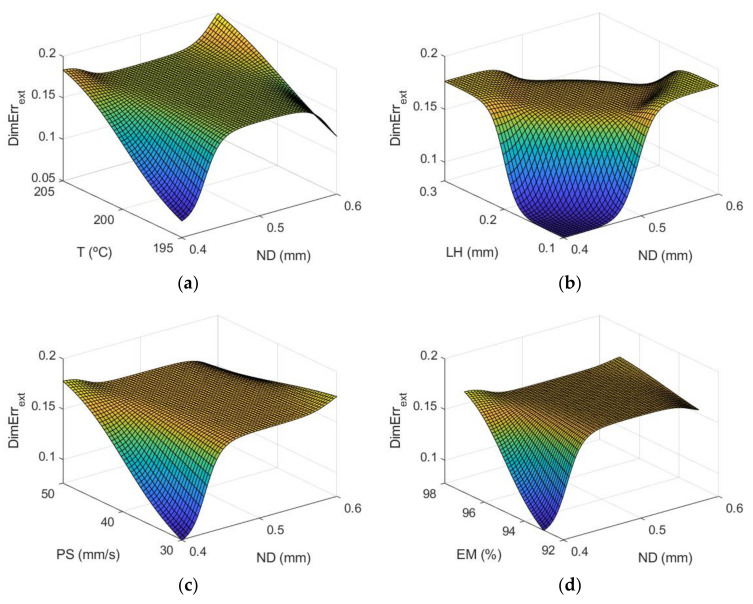

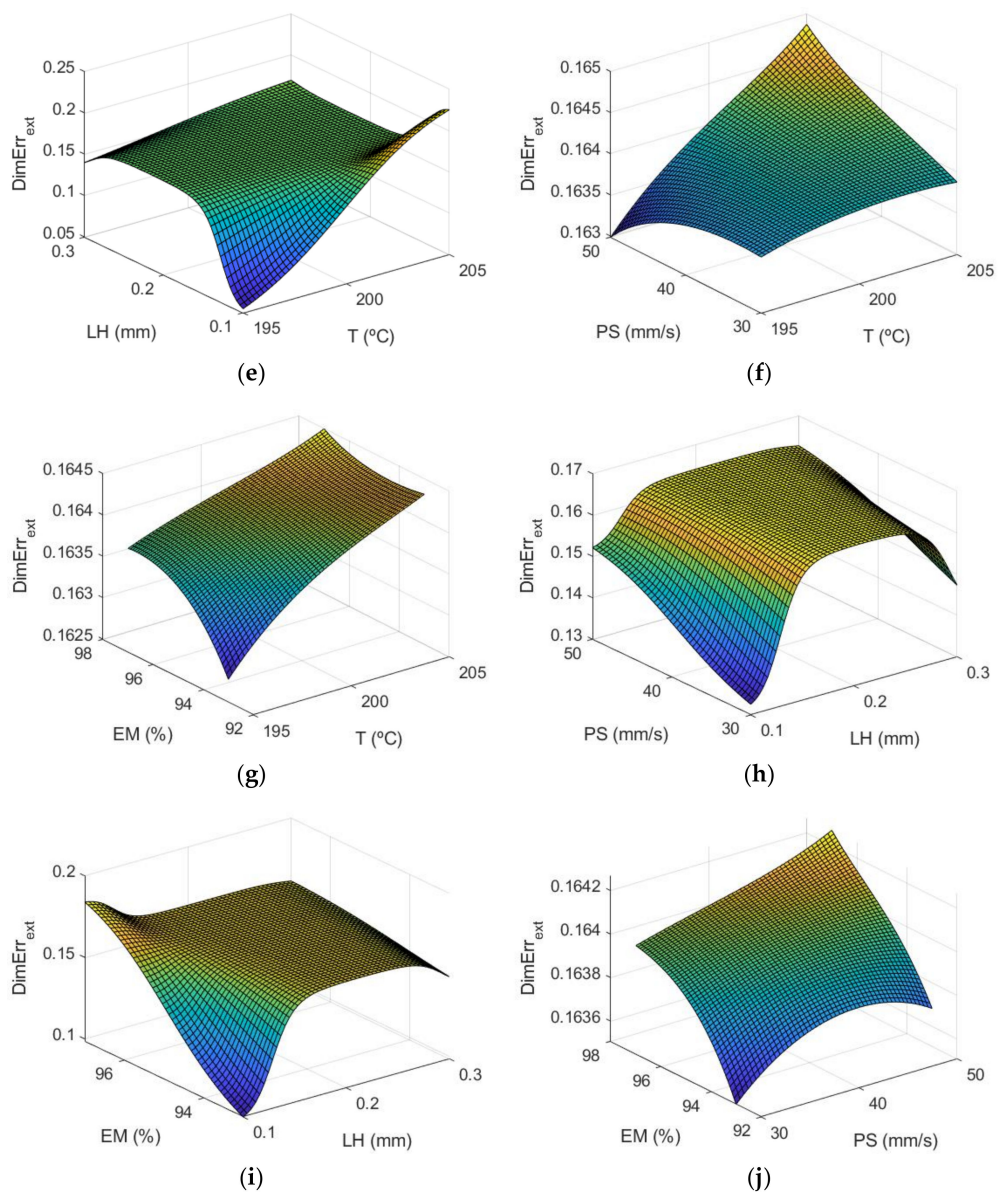
Response surfaces of dimensional error (mm) using the ANFIS for the case of the outer layer of the manufactured prototypes: (**a**) ND and T; (**b**) ND and LH; (**c**) ND and PS; (**d**) ND and EM; (**e**) T and LH; (**f**) T and PS; (**g**) T and EM; (**h**) LH and PS; (**i**) LH and EM; and (**j**) PS and EM.

**Figure 7:**
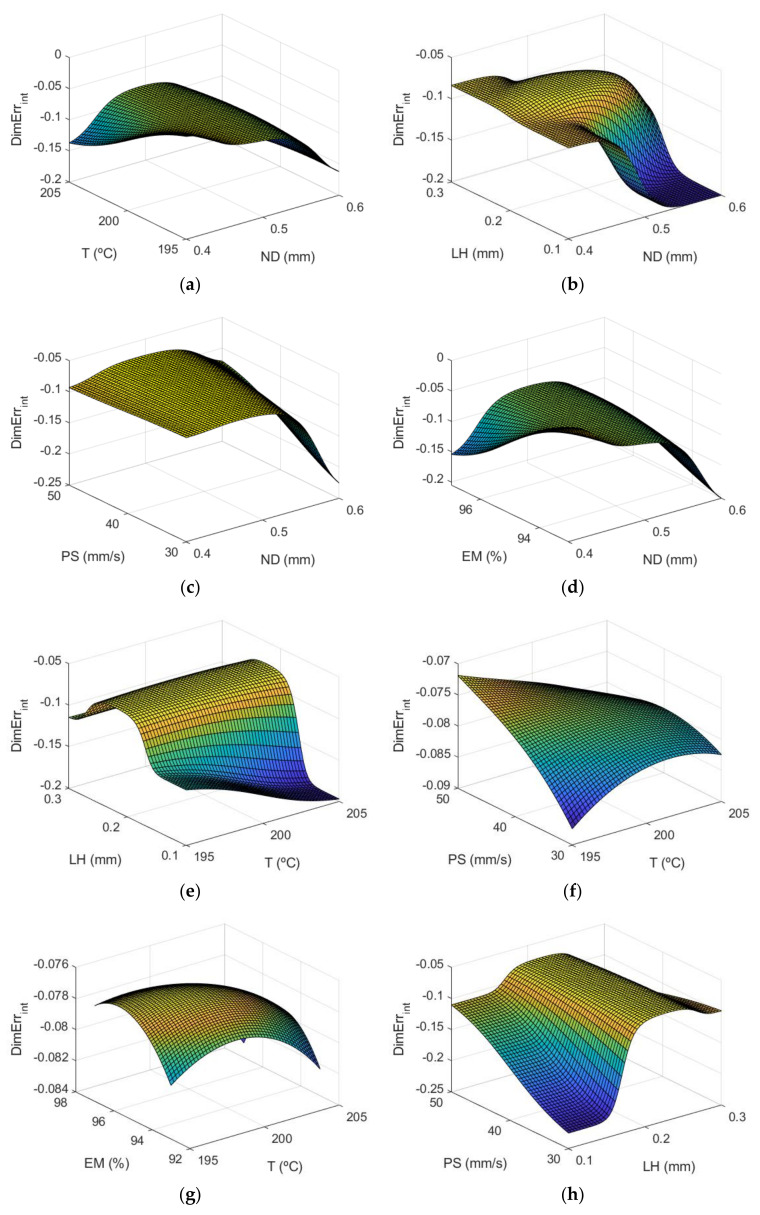

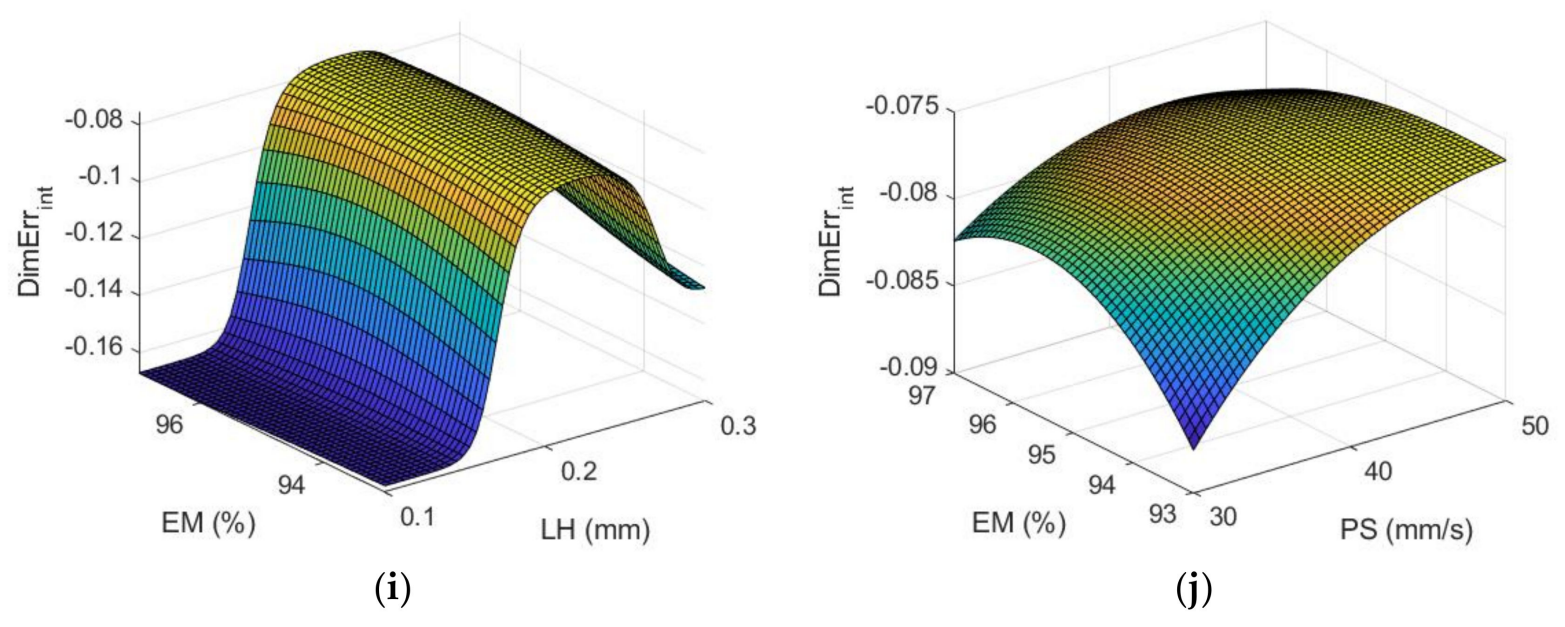
Response surfaces of dimensional error (mm) using the ANFIS for the case of the inner layer of the manufactured prototypes: (**a**) ND and T; (**b**) ND and LH; (**c**) ND and PS; (**d**) ND and EM; (**e**) T and LH; (**f**) T and PS; (**g**) T and EM; (**h**) LH and PS; (**i**) LH and EM; and (**j**) PS and EM.

Figure 6 shows the response surfaces by using the ANFIS models for the case of the dimensional error in the outer surface of the manufactured parts and Figure 7 shows those obtained for the internal part of the prototypes.

Figure 7 corresponds to the response surfaces for dimensional error of the inner surfaces of the parts.

**Figure 8:**
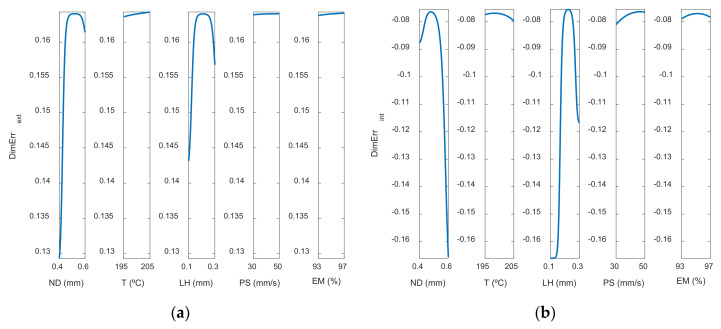
Main effects plots for (**a**) $Dimensional\;Error_{ext}\;\left(mm\right)$ and (**b**) $Dimensional\;Error_{int}\;\left(mm\right)$.

**Figure 9:**
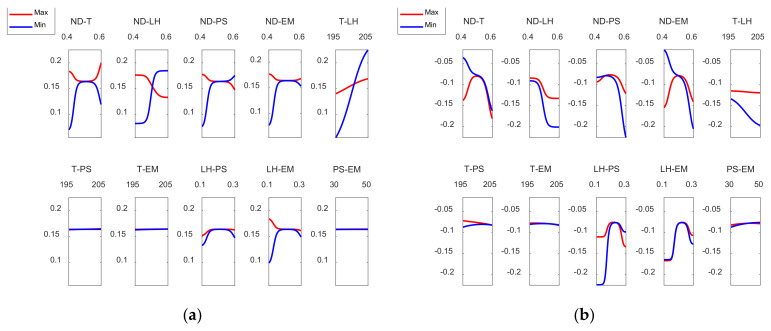
Inter action effects plots for (**a**) $Dimensional\;Error_{ext}\;\left(mm\right)$ and (**b**) $Dimensional\;Error_{int}\;\left(mm\right)$.

The most influential parameters on dimensional error are nozzle diameter and layer height. This is also observed in Figure 8 (main effects plots), and in Figure 9 (interaction effects plots).

Figure 8 depicts the main effect plots for both (a) the outer and (b) the inner zones of the manufactured prototypes. In these plots is shown the variation of each variable between its maximum and minimum levels, when all of the other factors are held at their average level.

As can be observed, the main parameters that affect the dimensional error are ND and LH, where the rest of the input variables have less influence on the dimensional error. An increase in nozzle diameter leads to an increase in dimensional error. In the outer surface of the parts, high nozzle diameter of 0.6 mm leads to high dimensional error. The measured diameter is higher than the theoretical one. In the inner surface of the parts, high nozzle diameter of 0.6 mm produces high dimensional error. In this case, since dimensional error values are negative, the measured diameter is lower than the theoretical one. This suggests that, when high nozzle diameter is employed, there is an excess of material. Dimensional error increases noticeably when layer height increases from 0.1 mm to 0.2 mm, but then decreases slightly if layer height of 0.3 mm is selected. Thus, low layer height of 0.1 mm is recommended in order to obtain low dimensional error. These results are in accordance with Nancharaiah et al. [7] regarding layer height. However, they recommended large bead width in order to reduce dimensional error. Spindola Filho et al. [15] found that print speed followed by layer height and the interaction between fill density and printing temperature (confounded with the interaction between print speed and layer height) were the most influential terms on dimensional error in ABS parts.

Figure 9 shows the interaction effects plot for the case of the dimensional error in both the outer and the inner zones of the prototypes.

As Figure 9a shows, in order to reduce the dimensional error, on the external surface, ND should be kept at its lowest level and at the same time T, LH, PS and EM should be kept at their minimum levels. On the other hand, the interaction between T and LH suggests that for low temperature values, LH should be at its minimum value to minimize the dimensional error. With regard to LH and EM, LH should be kept at its minimum level and the same is applicable to EM. In the case of Figure 9b, dimensional errors inside are mostly negative, since the measured diameter is lower than the nominal (theoretical) diameter. In this case, it is still interesting to keep ND at its lowest value and for T and EM to be at the minimum values. It does not matter that LH or PS are at minimum or maximum values while ND remains at its lowest level. In the case of the T-LH interaction, T should be kept at its minimum value. The effect of LH while ND is kept at the minimum value is not significant. However, if ND rises, LH should be at the maximum level, in order to minimize the dimensional error inside the manufactured parts. In the case of the interaction between LH and PS, for low LH values, it is interesting to keep PS at high values. On the other hand, for high LH values PS should be kept at low values. The rest of the interactions has less influence.

### 3.2. Models for Concentricity

**Figure 10:**
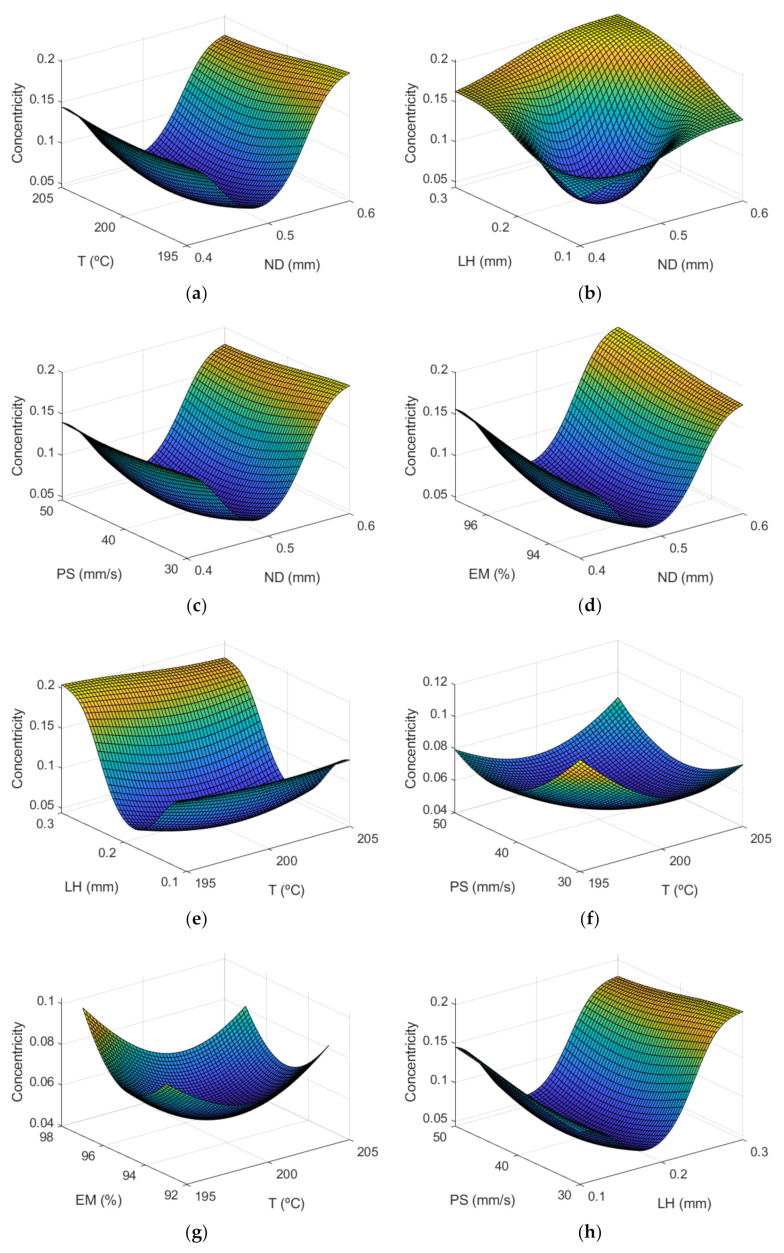

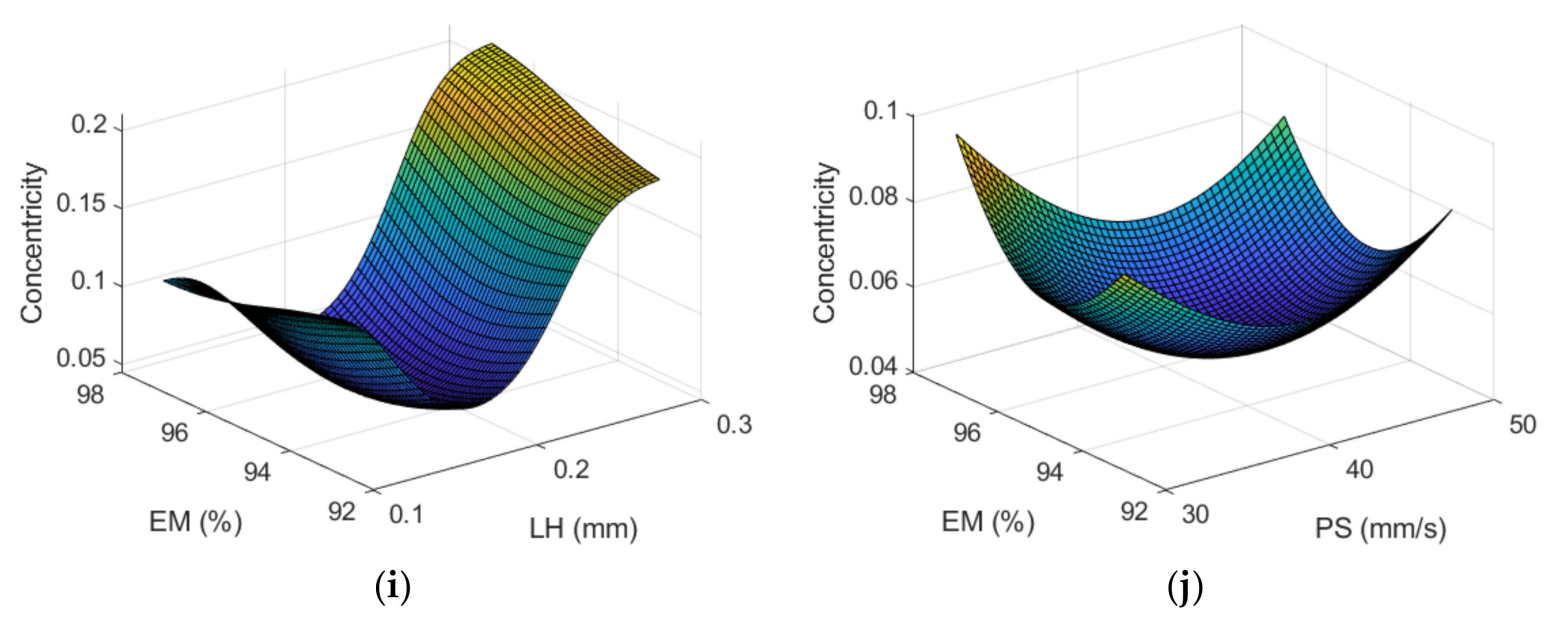
Surface response of concentricity (mm) using the ANFIS for the case of the manufactured prototypes: (**a**) ND and T; (**b**) ND and LH; (**c**) ND and PS; (**d**) ND and EM; (**e**) T and LH; (**f**) T and PS; (**g**) T and EM; (**h**) LH and PS; (**i**) LH and EM; and (**j**) PS and EM.

Figure 10 shows the response surface for the case of the concentricity.

As can be observed, the parameters that have the most effect are LH and ND. Conversely, Spindola Filho et al. [15] found that most influential parameter on concentricity was print speed. However, they did not vary the nozzle diameter and they employed higher layer height values between 0.2 and 0.4 mm.

**Figure 11:**
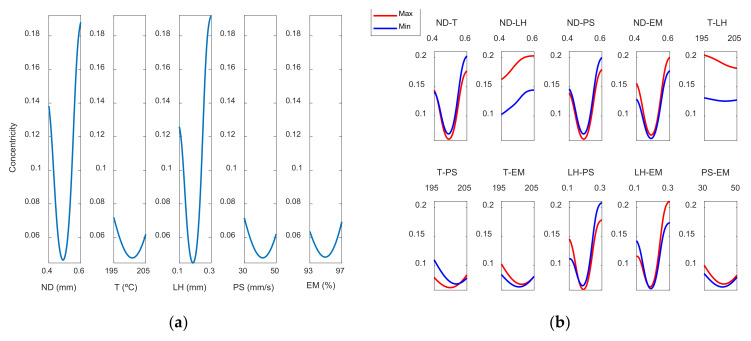
(**a**) Main effects plots and (**b**) interaction effects plots for Concentricity.

Finally, Figure 11 shows both the main and the interaction effects plot for the input variables under study.

Lowest concentricity error is obtained when medium nozzle diameter of 0.5 mm and medium layer height of 0.2 mm are selected. However, those conditions provide highest dimensional error (see Figure 8).

It may be noted that the interaction between variables is less than that obtained in the case of the dimensional error.

### 3.3. Models for Roundness Errors

**Figure 12:**
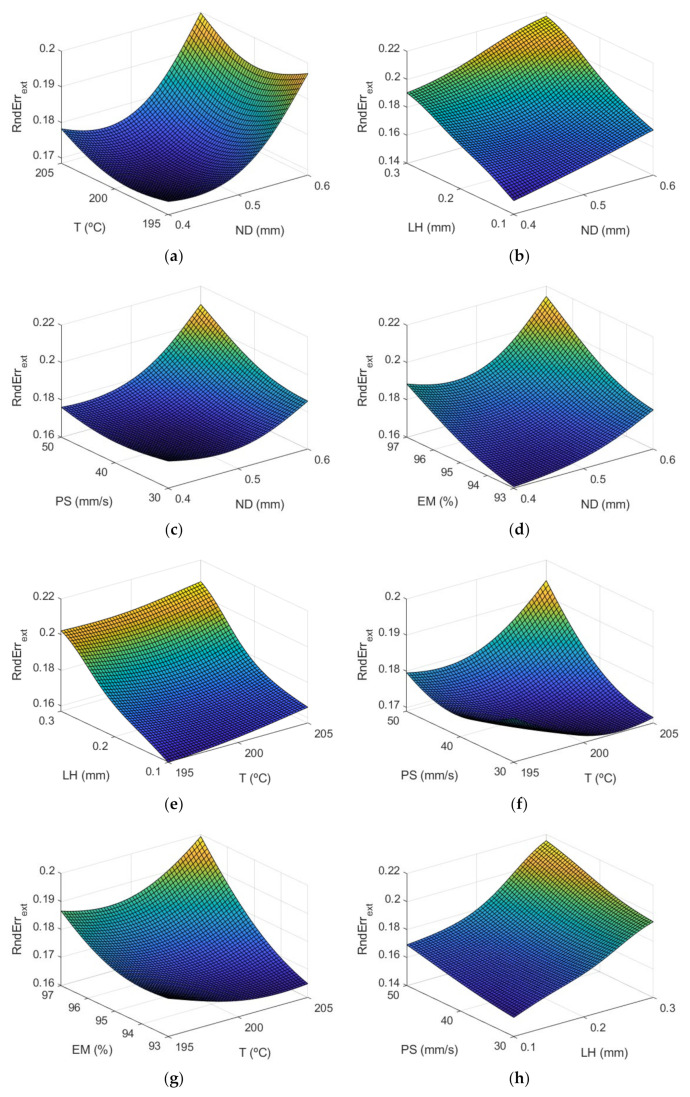

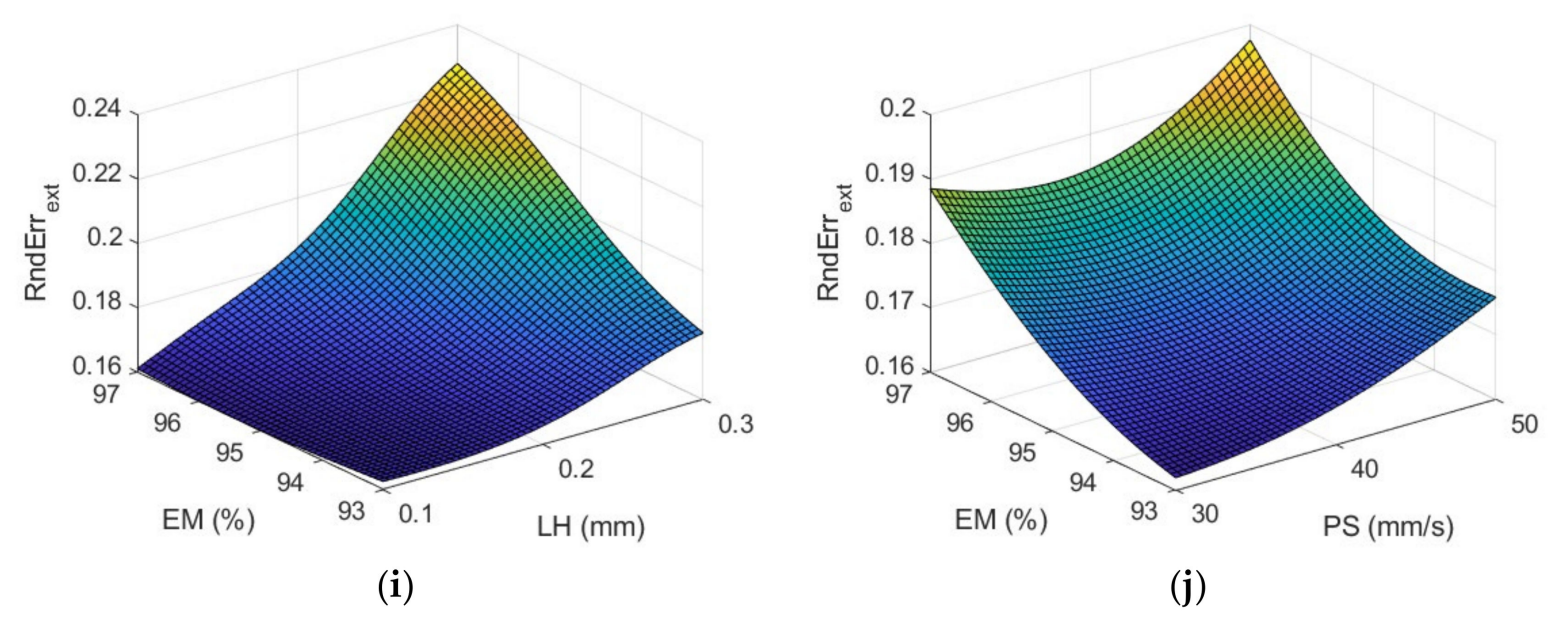
Response surfaces of roundness (mm) using the ANFIS for the case of the outer layer of the manufactured prototypes: (**a**) ND and T; (**b**) ND and LH; (**c**) ND and PS; (**d**) ND and EM; (**e**) T and LH; (**f**) T and PS; (**g**) T and EM; (**h**) LH and PS; (**i**) LH and EM; and (**j**) PS and EM.

**Figure 13:**
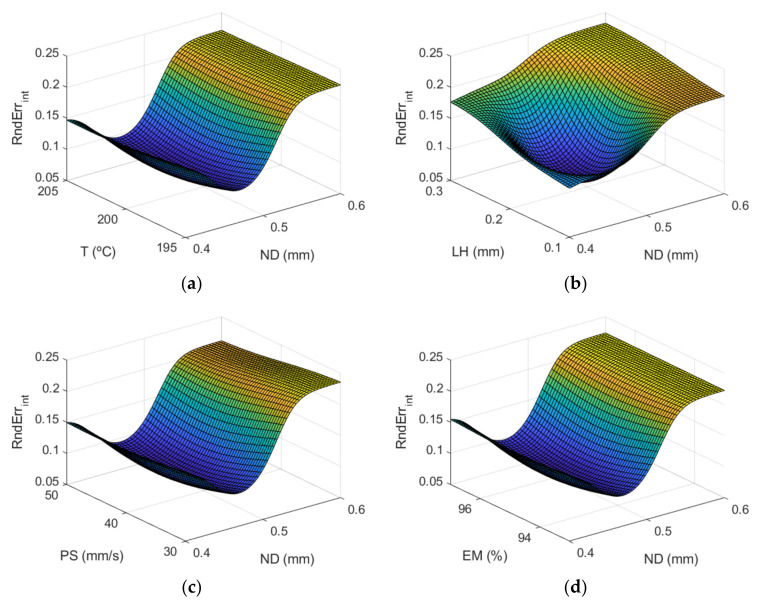

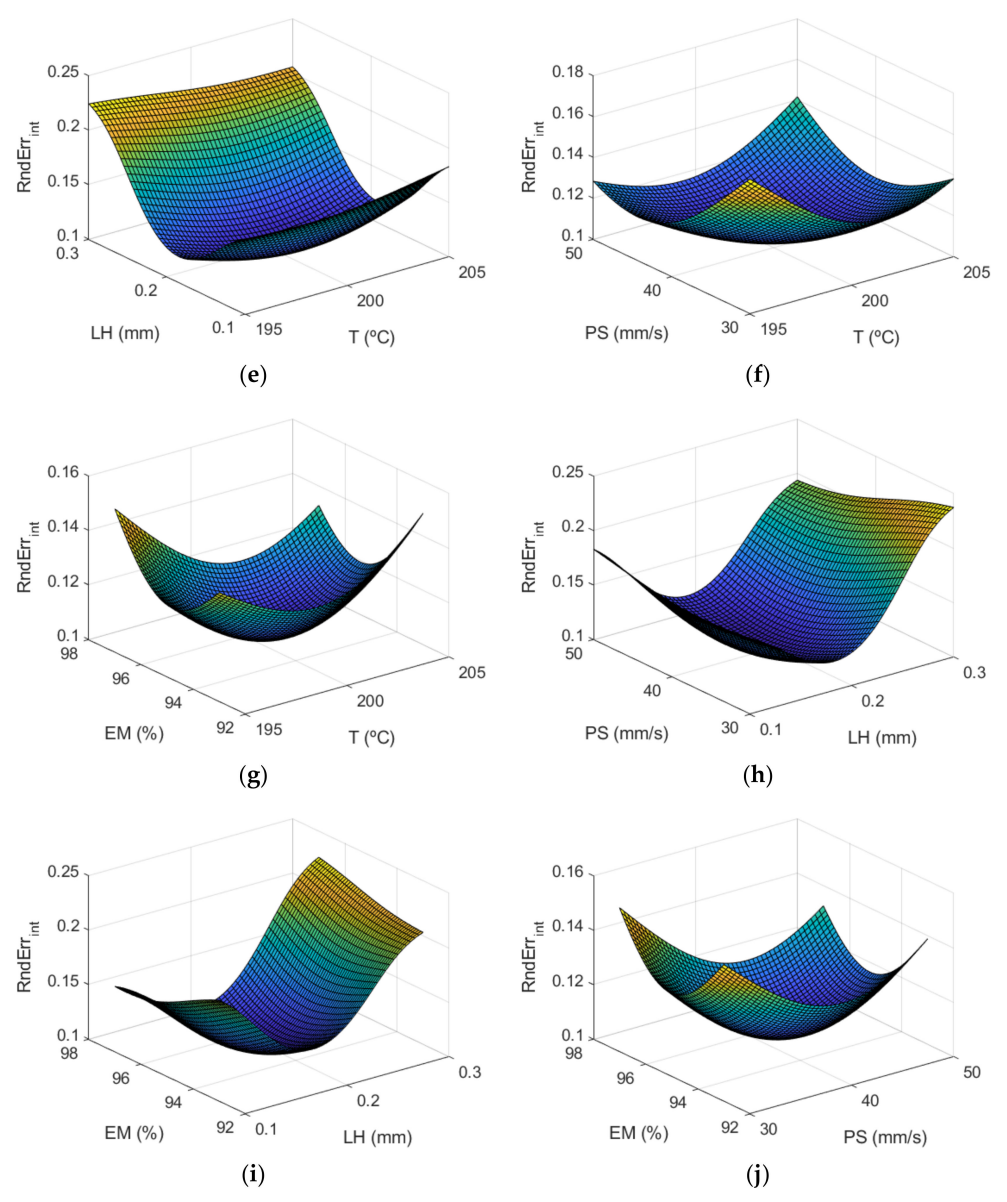
Surface response of roundness (mm) using the ANFIS for the case of the inner layer of the manufactured prototypes: (**a**) ND and T; (**b**) ND and LH; (**c**) ND and PS; (**d**) ND and EM; (**e**) T and LH; (**f**) T and PS; (**g**) T and EM; (**h**) LH and PS; (**i**) LH and EM; and (**j**) PS and EM.

Figure 12 and Figure 13 show the response surfaces for the case of the roundness by using the ANFIS models, in the outer and inner areas, respectively.

**Figure 14:**
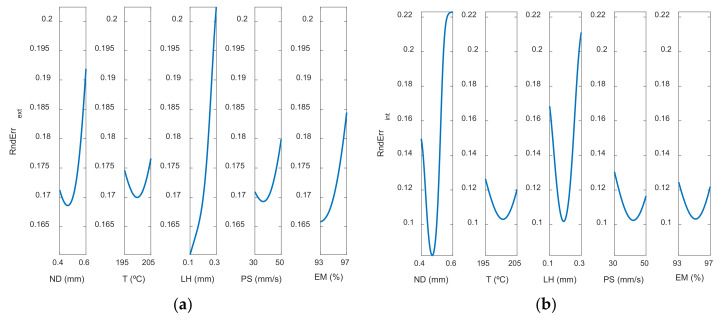
Main effects plots for (**a**) ${\mathrm{Roundness}\;}_{ext}\;\left(mm\right)$ and (**b**) ${\mathrm{Roundness}}_{int}\;\left(mm\right)$.

**Figure 15:**
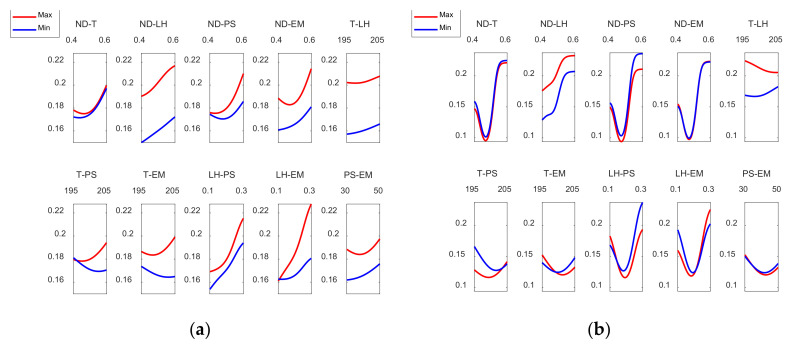
Interaction effects plots for (**a**) ${\mathrm{Roundness}\;}_{ext}\;\left(mm\right)$ and (**b**) ${\mathrm{Roundness}}_{int}\;\left(mm\right)$.

The information included in Figure 12 and Figure 13 is explained in more detail in Figure 14 and Figure 15. Figure 14 shows the main effect plots for the case of the roundness on both surfaces.

It may be seen that the parameters that most affect roundness are LH and ND. Roundness error decreases slightly when nozzle diameter increases from 0.4 to 0.5 mm but increases noticeably when nozzle diameter of 0.6 mm is selected. Highest roundness error is found when layer height of 0.3 mm is selected. To sum up, high layer height of 0.3 mm and high nozzle diameter of 0.6 mm are not recommended, since they provide high roughness error, high concentricity error and high dimensional error. Accordingly, as for the reduction of cylindricity of the printed parts, Maurya et al. [10] recommended low layer thickness of 100 μm. Conversely, Spindola Filho et al. [15] observed that print speed was the most influential factor on the cylindricity of ABS printed parts.

Finally, Figure 15 shows the interaction effects plot for roundness for the case of the outer and inner zones, respectively.

**Figure 16:**
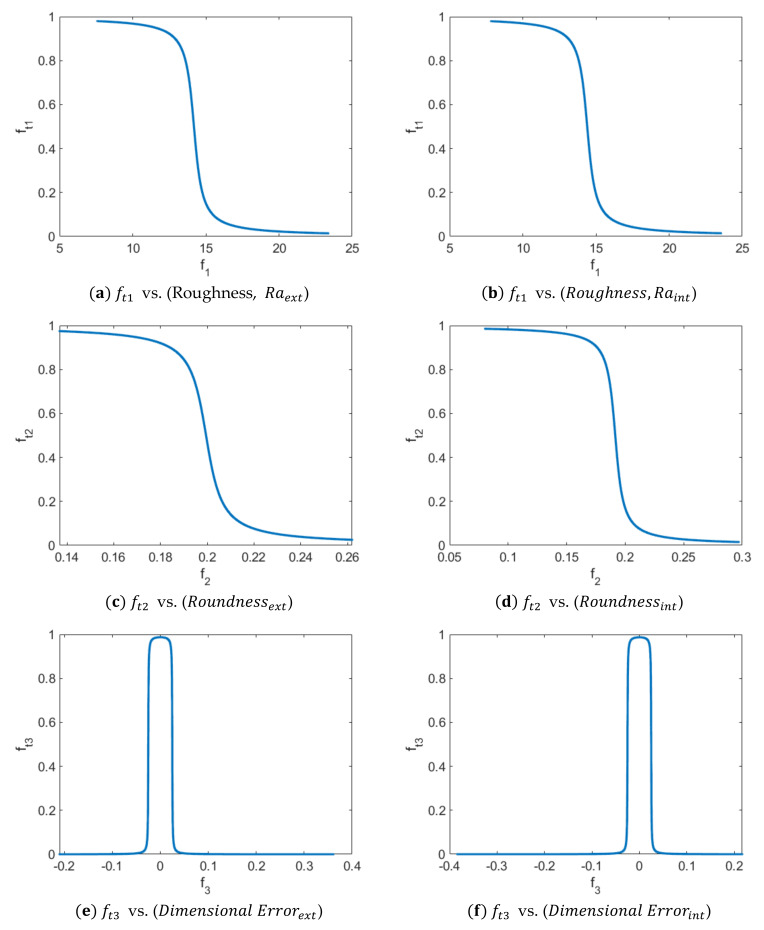
Transformation of the values of the response functions obtained by using the ANFIS and the desirability function proposed by Luis-Pérez [31] for the case of the outer surface (a–c) and for the case of the inner surface (d–e): $\left(a\right)\;f_{t1}$ vs. (Roughness, $Ra_{ext}$), $\left(b\right)\;f_{t2}$ vs. ($Roundness_{ext}$), $\left(c\right)\;f_{t3}$ vs. ($Dimensional\;Error_{ext}$), $\left(d\right)\;f_{t2}$ vs. ($Roundness_{int}$), $\left(e\right)\;f_{t2}$ vs. ($Roundness_{int}$), and $\left(f\right)\;f_{t3}$ vs. ($Dimensional\;Error_{int}$).

Figure 16a shows that ND should be kept at its lowest value to minimize external roundness and that the preferred variation for T, LH, and EM is the minimum value. Likewise, the interaction between T and LH, suggests that both the temperature and the layer height are kept at their minimum values. A contrary behavior is observed in the T-PS and T-EM interactions where the temperature should be at the highest level and PS and EM should be kept at their lowest levels. Dealing with the interaction LH-PS, it can be pointed out that LH should be at its lowest level and the same is applicable to PS. Moreover, in the interaction between LH and EM, if LH is set to its minimum, the level of EM does not matter. However, if LH rises EM should remain at its lowest level. On the other hand, Figure 16b shows that, in order to minimize internal roundness, ND should be close to its minimum value, in this case it does not matter if T, PS and EM are at the maximum or minimum values. In the case of the interaction between ND and LH, it can be observed that both ND and LH should be kept at their lowest values. Similarly, in the T-LH interaction it can be observed that both the temperature and LH should be kept at their lowest values.

### 3.4. Multiobjetive Optimization

In order to determine a set of values that simultaneously optimize roughness, roundness and dimensional error, a multi-objective optimization has been carried out following the methodology shown in the research work by Luis-Pérez [31]. For this purpose, the modeling obtained with the ANFIS for the response variables under study along with the desirability function proposed by [31] is used for optimization. The variables to be optimized have been selected as the surface roughness, characterized by the arithmetic mean roughness (Ra), which was already studied in a previous work carried out by the authors of this manuscript. Therefore, the ANFIS models developed in this present study for roundness and dimensional error are employed, as well as the ANFIS developed for Ra in [30].

The multi-objective optimization study has been carried out for the five input variables and three cases have been considered as output variables to be optimized: (a) Minimize (Ra and Roundness) and at the same time keep $\left|Dimensional\;Error\right|\leq 0.01$, in the outer zone; (b) Minimize (Ra and Roundness) and at the same time keep $\left|Dimensional\;Error\right|\leq 0.01$ in the inner zone and (c) simultaneously optimize the outer and inner zone. That is, minimize (Ra and Roundness) (for both zones) and at the same time keep $\left|Dimensional\;Error\right|\leq 0.01$, for both the outer zone and the inner zone.

Figure 16 shows the transformation of the values of the response functions obtained by using the ANFIS (for Ra, Roundness and Dimensional Error) and the desirability function proposed by Luis- Pérez [31] for the case of the outer surface (a–c) and for the case of the inner surface (e–f).

The values used to represent the functions shown in Figure 16 are those obtained after observing convergence in the values provided by the desirability function for both the outer zone and the inner zone. Table 3 shows the results of the multi-objective optimization for case a) in which minimize (Ra and Roundness) and $\left|Dimensional\;Error\right|\leq 0.01$, for the outer surface of the manufactured parts. On the other hand, Table 4 shows the results of the multi-objective optimization: Minimize (Ra & Roundness) and $\left|Dimensional\;Error\right|\leq 0.01$ for the inner surface of the manufactured parts and finally Table 5 shows the results obtained for the case of the simultaneous optimization of the outer and inner surface.

As can be observed in Table 3, it is shown that the preferred values to simultaneously optimize roughness and roundness as well as dimensional error are those in which ND and LH are kept at their lower levels and PS and EM are also close to the lower values of the input variables, within the range of the values studied. On the other hand, the working temperature should be increased to the highest level. Other solutions were found with slightly lower desirability values, for example 0.9771, in which a low temperature of 195 °C is recommended, with the rest of the variables in their low values or similar, except for the extrusion multiplier which should be around 95%. Low temperature is recommended for the inner surface (Table 4) and for the simultaneous optimization of both surfaces (Table 5).

On the other hand, Table 4 shows that, similarly to results obtained in Table 3, ND and LH should be kept at their minimum values and PS and EM should be slightly increased in relation to the previous case. On the other hand, the temperature value, in the case of the interior zone, should be selected close to the minimum value selected for experimentation.

Finally, Table 5 shows the simultaneous optimization of roughness, roundness, and dimensional error for the case of both inner and outer surfaces (that is, six response variables). In this case, ND and LH should be kept at their minimum value, T should be close to its low value, while PS and EM should be close to their medium value. As was previously observed (Figure 8, Figure 11, and Figure 14), high nozzle diameter and high layer height are not recommended if dimensional and form errors are to be minimized.

## 4. Conclusions

In this present research study, the main factors that affect the dimensional accuracy and form error of hemispherical cups 3D printed in PLA using the FFF technology have been examined. Different experimental tests were carried out according to a fractional factorial design of experiments (DOE).

Modeling has been carried out using ANFIS to obtain response models for dimensional error, roundness and concentricity of the hemispherical cups. It was observed that all of the responses, namely dimensional error, roundness error, and concentricity depend mainly on nozzle diameter and layer height (considering both the outer and the inner surfaces of the parts). High nozzle diameter of 0.6 mm and high layer height of 0.3 mm are not recommended due to the fact that they increase dimensional and form errors.

The models were used together with a desirability function to obtain the input parameters that simultaneously optimize surface finish, roundness and dimensional error. This has been carried out both independently, for the outer surface and the inner surface of the manufactured parts, as well as for simultaneous optimization (that is, with the results obtained for the outer and the inner zones of the prototypes).

In the range of values and parts analyzed in this study, it has been found that in the first and second cases (outer and inner surface, respectively) layer height and nozzle diameter should be kept at their lower values (0.1 mm and 0.4 mm, respectively) and also print speed and extrusion multiplier should be kept, in both cases, at values close to the lowest ones (30 mm/s and 93%). Regarding the first case, which is related to the outer surface of the parts, unlike the other two cases, temperature should be kept at the maximum value, 205 °C. However, other solutions were found for the outer surface with slightly lower desirability values, in which a low temperature of 195 °C is recommended, with the rest of the variables are in their low values or similar (except for the extrusion multiplier, which should be around 95%).

Regarding the third case, simultaneous optimization (inner-outer), nozzle diameter and layer height, as well as temperature, should be kept at the lowest value of the DOE (0.4 mm, 0.1 mm and 195 °C respectively). Meanwhile, print speed and extrusion multiplier should be approximately at their central value (40 mm/s and 95%).

## Figures and Tables

**Table 1:** Outputs.

Outputs (Int/Ext)		
Dimensional error	Roundness	Concentricity
(Dim Err, mm)	(Rnd, mm)	(Con, mm)

**Table 2:** Form errors (dimensional error, roundness) and concentricity.

						External		Internal		Concentricity
Exp	ND	T	LH	PS	EM	Dim Err	Rnd	Dim Err	Rnd	Con
						(mm)	(mm)	(mm)	(mm)	(mm)
1	0.4	195	0.1	30	97	0.084	0.137	−0.161	0.086	0.098
2	0.6	195	0.1	30	93	0.135	0.153	−0.384	0.201	0.125
3	0.4	205	0.1	30	93	−0.018	0.143	−0.090	0.089	0.093
4	0.6	205	0.1	30	97	0.243	0.137	−0.229	0.165	0.051
5	0.4	195	0.3	30	93	0.153	0.153	−0.112	0.190	0.120
6	0.6	195	0.3	30	97	0.198	0.229	−0.137	0.297	0.272
7	0.4	205	0.3	30	97	0.044	0.222	0.156	0.162	0.176
8	0.6	205	0.3	30	93	0.146	0.164	−0.178	0.235	0.182
9	0.4	195	0.1	50	93	−0.210	0.141	0.217	0.137	0.083
10	0.6	195	0.1	50	97	0.057	0.149	−0.057	0.167	0.127
11	0.4	205	0.1	50	97	0.361	0.147	−0.26	0.129	0.100
12	0.6	205	0.1	50	93	0.290	0.209	−0.184	0.270	0.202
13	0.4	195	0.3	50	97	0.188	0.195	−0.187	0.163	0.158
14	0.6	195	0.3	50	93	−0.025	0.206	−0.053	0.194	0.175
15	0.4	205	0.3	50	93	0.314	0.161	−0.200	0.129	0.140
16	0.6	205	0.3	50	97	0.190	0.262	−0.153	0.214	0.190
17-1	0.5	200	0.2	40	95	0.145	0.180	−0.074	0.110	0.053
17-2	0.5	200	0.2	40	95	0.234	0.175	−0.042	0.101	0.066
17-3	0.5	200	0.2	40	95	0.113	0.155	−0.115	0.099	0.026

**Table 3:** Multi-objective optimization results: minimize (Ra & Roundness) and $\left|Dimensional\;Error\right|\leq 0.01$, for the outer surface of the manufactured parts using the desirability function and the methodology proposed by Luis-Pérez [31].

Desirability Value	ND	T	LH	PS	EM	Ra (µm)	Roundness (mm)	Dimensional Error (mm)
0.9780	0.4000	205.0000	0.1000	31.0526	93.6316	8.3972	0.1460	−0.0054

**Table 4:** Multi-objective optimization results: minimize (Ra & Roundness) and $\left|Dimensional\;Error\right|\leq 0.01$, for the inner surface of the manufactured parts using the desirability function and the methodology proposed by Luis-Pérez [31].

Desirability Value	ND	T	LH	PS	EM	Ra (µm)	Roundness (mm)	Dimensional Error (mm)
0.9764	0.4000	195.5882	0.1000	39.4118	94.8824	10.4376	0.1267	−0.0010

**Table 5:** Multi-objective optimization results: minimize (Ra and Roundness) and $\left|Dimensional\;Error\right|\leq 0.01$, simultaneously for the inner and the outer surfaces of the manufactured parts using the desirability function and the methodology proposed by Luis-Pérez [31].

Desirability Value	ND	T	LH	PS	EM		Ra (µm)	Roundness (mm)	Dimensional Error (mm)
						Ext.	9.2643	0.1476	−0.0048
0.9759	0.4000	197.6667	0.1000	42.0000	94.8667				
						Int.	10.4977	0.1320	−0.0022

## Data Availability

Not applicable.
